# Supplementary material for: An extended retinotopic map of mouse cortex
Source: eLife. 2017 Jan 6;6:e18372. doi: 10.7554/eLife.18372 (PMC5218535; doi:10.7554/eLife.18372)
Supplement: Supplementary file 1. — Example of derivation of a field sign map from altitude and azimuth maps, in HTML format. The same example is available as a Jupyter notebook at https://github.com/zhuangjun1981/retinotopic_mapping. DOI: http://dx.doi.org/10.7554/eLife.18372.027 [file elife-18372-supp1.htm]

retinotopic\_mapping\_example


In [1]:

```
import os
import retinotopic_mapping
import retinotopic_mapping.core.FileTools as ft
import retinotopic_mapping.RetinotopicMapping as rm
import tifffile as tf
import matplotlib.pyplot as plt

%matplotlib inline
```

In [2]:

```
# move to package example folder 

package_folder = retinotopic_mapping.__path__
example_folder = os.path.join(package_folder[0], 'examples')
os.chdir(example_folder)
```

# Reading example vasculature image¶

In [3]:

```
vasculature_map = tf.imread('example_vasculature_map.tif')
_ = plt.imshow(vasculature_map, cmap='gray', interpolation='nearest')
_ = plt.colorbar()
plt.show()
```

# Reading example retinotopic maps¶

In [4]:

```
altitude_map = tf.imread('example_altitude_map.tif')
azimuth_map = tf.imread('example_azimuth_map.tif')
altitude_power_map = tf.imread('example_altitude_power_map.tif')
azimuth_power_map = tf.imread('example_azimuth_power_map.tif')

f = plt.figure(figsize=(15, 12))
ax1 = f.add_subplot(221)
fig1 = ax1.imshow(altitude_map, vmin=-40, vmax=60, cmap='hsv', interpolation='nearest')
ax1.set_axis_off()
ax1.set_title('altitude map')
_ = f.colorbar(fig1)

ax2 = f.add_subplot(222)
fig2 = ax2.imshow(azimuth_map, vmin=0, vmax=120, cmap='hsv', interpolation='nearest')
ax2.set_axis_off()
ax2.set_title('azimuth map')
_ = f.colorbar(fig2)

ax3 = f.add_subplot(223)
fig3 = ax3.imshow(altitude_power_map, vmin=0, vmax=1, cmap='hot', interpolation='nearest')
ax3.set_axis_off()
ax3.set_title('altitude power map')
_ = f.colorbar(fig3)

ax4 = f.add_subplot(224)
fig4 = ax4.imshow(azimuth_power_map, vmin=0, vmax=1, cmap='hot', interpolation='nearest')
ax4.set_axis_off()
ax4.set_title('azimuth power map')
_ = f.colorbar(fig4)
```

# Defining image analysis parameters¶

#### This is probably the most important part of the whole analysis! The 14 parameters below define the final segmentation results.¶

- phaseMapFilterSigma: The sigma value (in pixels) of Gaussian filter for altitude and azimuth maps. FLOAT, default = 1.0, recommended range: [0.0, 2.0]. Large "phaseMapFilterSigma" gives you more patches. Small "phaseMapFilterSigma" gives you less patches.
- signMapFilterSigma: The sigma value (in pixels) of Gaussian filter for visual sign maps. FLOAT, default = 9.0, recommended range: [0.6, 10.0]. Large "signMapFilterSigma" gives you less patches. Small "signMapFilterSigma" gives you more patches.
- signMapThr: Threshold to binarize visual signmap. FLOAT, default = 0.35, recommended range: [0.2, 0.5], allowed range: [0, 1). Large signMapThr gives you fewer patches. Smaller signMapThr gives you more patches.
- closeIter: Binary close iteration for each raw patches. INT, default = 3. You do not want to change this parameter very often.
- openIter: Binary open iteration for each raw patches. INT, default = 3. You do not want to change this parameter very often
- dilationIter: Binary dilation iteration for each raw patches. INT, default = 15. You do not want to change this parameter very often.
- borderWidth: Pixel number between adjcent patches. INT, default = 1. You do not want to change this parameter very often.
- smallPatchThr: The patches with pixel number below smallPatchThr will be discarded. INT, default = 100. You do not want to change this parameter very often.
- eccMapFilterSigma: The sigma value of Gaussian filter for eccentricity maps. FLOAT, default = 10.0. You do not want to change this parameter very often.
- visualSpacePixelSize: The pixel size for patch to visual space mapping. FLOAT, default = 0.5. You do not want to change this parameter very often.
- visualSpaceCloseIter: The binary iteration for visual space coverage for each patch. INT, default = 15. You do not want to change this parameter very often.
- splitLocalMinCutStep: The step width for detecting number of local minimums during spliting. The local minimums detected will be used as marker in the following open cv watershed segmentation. FLOAT, default = 5.0, recommend range: [0.5, 15.0]. Small "splitLocalMinCutStep" will make it more likely to split but into less sub patches. Large "splitLocalMinCutStep" will make it less likely to split but into more sub patches.
- splitOverlapThr: Patches with overlap ration larger than this value will go through the split procedure. FLOAT, default = 1.1, recommend range: [1.0, 1.2], should be larger than 1.0. Small "splitOverlapThr" will split more patches. Large "splitOverlapThr" will split less patches.
- mergeOverlapThr: Considering a patch pair (A and B) with same sign, A has visual coverage a deg2 and B has visual coverage b deg2 and the overlaping visual coverage between this pair is c deg2. Then if (c/a < "mergeOverlapThr") and (c/b < "mergeOverlapThr"), these two patches will be merged. FLOAT, default = 0.1, recommend range: [0.0, 0.2], should be smaller than 1.0. Small "mergeOverlapThr" will merge less patches. Large "mergeOverlapThr" will merge more patches.

In [5]:

```
params = {
          'phaseMapFilterSigma': 0.5,
          'signMapFilterSigma': 8.,
          'signMapThr': 0.4,
          'eccMapFilterSigma': 15.0,
          'splitLocalMinCutStep': 5.,
          'closeIter': 3,
          'openIter': 3,
          'dilationIter': 15,
          'borderWidth': 1,
          'smallPatchThr': 100,
          'visualSpacePixelSize': 0.5,
          'visualSpaceCloseIter': 15,
          'splitOverlapThr': 1.1,
          'mergeOverlapThr': 0.1
          }
```

# Creating the RetinotopicMappingTrail object¶

In [6]:

```
trial = rm.RetinotopicMappingTrial(altPosMap=altitude_map,
                                   aziPosMap=azimuth_map,
                                   altPowerMap=altitude_power_map,
                                   aziPowerMap=azimuth_power_map,
                                   vasculatureMap=vasculature_map,
                                   mouseID='test',
                                   dateRecorded='160612',
                                   comments='This is an example.',
                                   params=params)
```

In [7]:

```
print trial
```

```
A retinotopic mapping trial: 160612_Mtest
```

# Generating visual sign map¶

In [8]:

```
_ = trial._getSignMap(isPlot=True)
plt.show()
```

# Binarizing filtered visual signmap¶

In [9]:

```
_ = trial._getRawPatchMap(isPlot=True)
plt.show()
```

# Generating raw patches¶

In [10]:

```
_ = trial._getRawPatches(isPlot=True)
plt.show()
```

# Generating determinant map¶

In [11]:

```
_ = trial._getDeterminantMap(isPlot=True)
plt.show()
```

# Generating eccentricity map for each patch¶

In [12]:

```
_ = trial._getEccentricityMap(isPlot=True)
plt.show()
```

# Splitting overlapping patches¶

In [13]:

```
_ = trial._splitPatches(isPlot=True)
plt.show()
```

```
E:\data\python_packages\retinotopic_mapping\retinotopic_mapping\RetinotopicMapping.py:2670: VisibleDeprecationWarning: using a non-integer number instead of an integer will result in an error in the future
  np.ceil((aziRange[1]-aziRange[0]) / pixelSize)))
```

```
patch06 AU=387.25  AS=379.023035519  ratio=0.978755417738
patch05 AU=781.0  AS=751.161853989  ratio=0.961794947489
patch09 AU=124.75  AS=115.714161245  ratio=0.927568426815
patch08 AU=577.75  AS=555.873222612  ratio=0.962134526373
patch04 AU=907.5  AS=949.386748708  ratio=1.04615619692
patch12 AU=92.25  AS=82.7706059634  ratio=0.897242341066
patch13 AU=56.25  AS=51.598631273  ratio=0.917309000408
patch07 AU=333.0  AS=315.801067694  ratio=0.948351554637
patch11 AU=74.25  AS=80.4597385201  ratio=1.08363284202
patch01 AU=3611.25  AS=4029.63594829  ratio=1.11585626813
2 local minuma were found!!!
```

```
E:\data\python_packages\retinotopic_mapping\retinotopic_mapping\RetinotopicMapping.py:395: RuntimeWarning: invalid value encountered in less_equal
  marker[eccMap2 <= (currThr)] = 1
```

```
patch10 AU=109.5  AS=105.310979046  ratio=0.961744100879
patch03 AU=1916.0  AS=1635.29604889  ratio=0.85349480631
patch02 AU=1106.75  AS=1206.21250126  ratio=1.08986898691
```

# Merging non-overlapping patches¶

In [14]:

```
_ = trial._mergePatches(isPlot=True)
plt.show()
```

```
merge iteration: 1
merging: patch01.1 & patch13, overlap ratio: 0.0888888888889
merge iteration: 2
```

## Note:¶

### The methods used from cell 8 to cell 14 are protected by leading underscore! Here they were used separately to show every single image analysis step. In real life, the steps from cell 8 to cell 14 can be consolidated into one single method:¶

`trial.processTrial(isPlot=True)`

# Plotting results¶

In [15]:

```
_ = trial.plotFinalPatchBorders2()
plt.show()
```

# Annotating segmented patches¶

In [20]:

```
names = [
         ['patch01', 'V1'],
         ['patch02', 'PM'],
         ['patch03', 'RL'],
         ['patch04', 'P'],
         ['patch05', 'LM'],
         ['patch06', 'AM'],
         ['patch07', 'LI'],
         ['patch08', 'MMA'],
         ['patch09', 'AL'],
         ['patch10', 'RLL'],
         ['patch11', 'LLA'],
#          ['patch12', 'MMP'],
         ['patch13', 'MMP']
         ]

finalPatchesMarked = dict(trial.finalPatches)

for i, namePair in enumerate(names):
    currPatch = finalPatchesMarked.pop(namePair[0])
    newPatchDict = {namePair[1]:currPatch}
    finalPatchesMarked.update(newPatchDict)
    
trial.finalPatchesMarked = finalPatchesMarked
```

# Ploting final results¶

In [21]:

```
_ = trial.plotFinalPatchBorders2()
plt.show()
```

# Generating dictionary for saving¶

In [22]:

```
trialDict = trial.generateTrialDict()
trialDict.keys()
```

Out[22]:

```
['eccentricityMapf',
 'mouseID',
 'aziPowerMap',
 'altPosMapf',
 'altPowerMap',
 'aziPowerMapf',
 'comments',
 'aziPosMap',
 'altPosMap',
 'finalPatchesMarked',
 'signMapf',
 'params',
 'finalPatches',
 'vasculatureMap',
 'signMap',
 'dateRecorded',
 'altPowerMapf',
 'rawPatchMap',
 'aziPosMapf']
```

# Saving results¶

In [19]:

```
# ft.saveFile("path_to_save", trialDict)
```
